# Thomas Harrison Burder

**Published:** 1844-01

**Authors:** 


### OBITUARY.

### Sketch of the Life and Character of the late Thomas Harrison Burder, M.D.

The subject of this brief notice died at Tunbridge Wells, on the 16th of August, 1843, in his fifty-fourth year. His ancestors, for several generations, had been remarkable for simplicity, benevolence, and wisdom; and his father, the Rev. George Burder, was one of the most distinguished Christian philanthropists of his day. Dr. Burder's life was marked by few incidents calculated to attract attention; but it presents an example of self-denying humanity as well as of conscientious devotion to professional duty, which ought not to pass unrecorded.

Dr. Burder studied medicine at Edinburgh with much assiduity, and obtained the friendship of some of the best and wisest men adorning that seat of learning: where also his characteristic benevolence was evinced by the faithful and almost brotherly interest which he took in the welfare and success of those students with whom he was on terms of intimacy. In 1812 he was elected to the office of President of the Medical Society, an appointment always indicative of merit in the holder. He left Edinburgh in 1815, and shortly afterwards commenced practice in London. He soon, also, connected himself with a large public dispensary, the duties of which he fulfilled with exemplary zeal and humanity. In 1827 he married and settled in Brunswick square; but his health proved unequal to the exertions which a steadily increasing professional reputation necessarily entailed.

His constitution was naturally susceptible; he had long been subject to attacks of dyspeptic headach, and had frequently suffered from mental application during a long and laborious course of study. A severe attack of headach thus produced at Edinburgh, treated by depletion, general and topical, supposed to have been excessive, increased his natural susceptibility to such a degree, that his subsequent life was but a long disease, confirmed perhaps or aggravated by perseverance in mental labour and excitement, and by a prepossession in favour of lowering treatment. The onerous nature of his duties in London brought on an increase of the pain and excitement of head. For these symptoms, general and local bleeding, murcurial purgatives, antimony, and low diet were considered necessary. Under this treatment the symptoms certainly subsided considerably, but the brain and nervous system remained in a state of augmented and extreme susceptibility.

On some recurrence of pain it was judged needful to shave the head. This happened to be done on a cold wet evening, under circumstances of great exhaustion, and the ordinary covering of a thin nightcap was alone worn during the ensuing night. On awakening from sleep a severe constrictive pain was felt over the whole head, attended with heat and tenderness of the scalp, throbbing of the temporal arteries, much cerebral excitement, and vomiting. An affection of the pericranium was now considered by him to have been ingrafted on cerebral excitement; but, after the superficial tenderness had subsided, the original affection still proved intractable.

Such was the severe and various suffering which so soon disqualified Dr. Burder for the arduous duties of medical practice in London. Any case involving more than ordinary anxiety aggravated the headach, and this anxiety was peculiarly felt by him in relation to diseases of the chest. The introduction of the new method of exploring pectoral diseases by auscultation was materially changing the aspect of this department of medicine, and a sense of duty urged him to become conversant with the indications afforded by the stethoscope; but he found the study beset with many difficulties, and a conscientious reluctance to undertake the treatment of pectoral complaints without possessing a more intimate acquaintance with the physical signs than his impaired constitutional energy would enable him promptly to obtain, at last determined him to leave London.

In the year 1834 Dr. Burder made a tour into Devonshire with advantage to his general health, but with no relief to his headach, which indeed was rather increased by the excitement of the journey. In the autumn he took up his abode at Tilford, near Farnham, and remained there four years. He enjoyed the quiet of this secluded spot, but the pain of head was not much lessened. Continued suffering, however, had not overcome his desire to be usefully employed; and having given a fair though unsuccessful trial to the plan of repose, in 1837 he settled at Tunbridge Wells, where the growing confidence of his professional brethren again placed his talents in request. But in the autumn of 1838 increasing headach obliged him to restrict his attentions to a limited number of patients at his own residence; and the corroding anxiety produced in his mind by hopeless cases, at last induced him to withdraw from practice altogether.

In the year 1840, after irritation of the intestines and other organs, accompanied with rheumatic pains, some œdema of the legs occurred, and his strength was considerably reduced. This state continued in the following year. The heart had also been supposed to be affected; but, on careful examination, no change in that organ could be detected. In the summer of 1842 an obstinate and prolonged attack of constipation, for which calomel, colocynth, and ultimately turpentine were administered, was followed by pain of the arms and muscular debility, rendering it difficult to button the coat or guide the hand in writing, yet without loss of feeling. The pulse conveyed to the touch the impression of a vessel supplied with blood, defective both in quantity and quality; which circumstance, and the presence of nervous symptoms resembling those commonly present in hysteria, led to the recommendation of iron as a remedy. The citrate was accordingly tried, but soon suspended in consequence of the patient's apprehension that it increased the headach. His general strength afterwards improved, but some weakness remained in the thumb and index-finger of the left hand. A visit to Brighton produced slight amendment, but he soon relapsed into a state of great weakness accompanied with intestinal torpor. During the early part of 1843 he perceptibly declined in strength, and in August was reduced to a state of extreme debility. On one occasion, asking for a looking-glass, and observing an aphthous appearance of the mouth, which, under the circumstances, was an ominous symptom, he calmly remarked, "I was quite right—this attack is fatal;" and within a few days afterwards made a peaceful exit from this scene of protracted suffering.

The post-mortem examination revealed no very remarkable traces of disease. There was considerable effusion beneath the arachnoid membrane of the brain and spinal cord, but perhaps not more than frequently occurs in debilitated subjects independently of disease, shortly before or even subsequently to dissolution. The brain was firm and natural, but deficient in blood. The lining membrane of the stomach near the cardia much injected. The lungs and liver healthy. The heart small and soft, but free from disease.

The question naturally arises, whether the protracted sufferings in the head and bowels were the result of susceptibility produced by depletion, or were occasioned by organic conditions, prevented, by active treatment, from inducing visible changes of structure. But in whatever manner this question may be solved, it is difficult to avoid the conclusion that health and life were sacrificed to inordinate intellectual exertion and moral sympathies, the influence of which no physical treatment could effectually control.

Dr. Burder's mental qualities were of a superior order. His accuracy in observing facts, and caution in deducing conclusions, are evinced in an interesting paper published by him in the 'London Medical and Physical Journal' for June, 1837, on the question of the cerebral origin of paraplegia; in which he endeavours to prove, as in cases of this kind of palsy ascribed to the brain, the spine was either found to be affected or left unexamined. A habit of exact discrimination rendered his practical opinions remarkably free from empiricism. He gave a careful consideration to the peculiarities of each patient's case, and habitually prescribed for the individual *variety*, not for a *species* or *genus* of disease. His communications in the 'Cyclopædia of Practical Medicine,' on the subjects of Headach and Jaundice, possess considerable merit; and the former essay is particularly valuable, among other reasons, by enforcing a truth too often overlooked, namely, that in many cases of headach apparently arising from indigestion, the stomach is *secondarily* affected, in consequence of undue exertion of the *brain*.

In evidence of his intellectual activity, it may be mentioned that even when increasing indisposition rendered it necessary for him to retire from practice, he contemplated delivering a series of lectures on the circumstances which should guide selection among medicines of analogous properties; justly regarding the neglect of such discrimination as one of the chief sources of medical failure, and one of the principal encouragements to that ignorant and bold empiricism by which the public are so dangerously deluded.

During his seclusion at Farnham he did not relinquish schemes of active usefulness; and, amongst other things, produced the 'Letters from a Senior to a Junior Physician,' which are distinguished by a spirit of Christian philanthropy combined with sound discretion.

In illustration of the simplicity and unaffectedness of manner which characterized Dr. Burder, it may be interesting to adduce his answer to the inquiry to what extent a medical practitioner might be justified in studying a manner best calculated to win the public confidence.

"Doubtless," he observes, "a confident manner and an oracular expression of opinion, superficial and inaccurate though it may be, obtain from the multitude an undue degree of respect *at first*; but the more modest and more highly qualified man will ultimately surmount any real or supposed external deficiencies; while every instance of gratifying confidence will do much to give a legitimately authoritative address. Close attention, unaffected interest and earnestness, careful investigation, and perfect candour and simplicity are qualities which almost every one can appreciate, and which will eventually command a more extensive influence than all the arts and manœuvres of self-confident boasters. At all events a man's natural manner is the best for himself, since it accords with his mind and character; and, being natural, will give a genuineness for which all the elegancies and studied doings of others could not compensate."

Dr. Burder's mind, kept vigilantly free from prepossessions and routine, was always disposed to yield an unprejudiced consideration to any suggestion offered in a philosophical spirit.

In his general demeanour, a manner peculiarly bland and courteous, harmonized with a graceful delicacy which shrunk from wounding the feelings of the humblest individual; whilst no amount of personal suffering suppressed the expansive zeal ever kindling within him to promote every benevolent scheme for the benefit of his fellow-men. Equally apt to applaud excellence and disinclined to censure imperfection, he was never known to sully the fair fame of a competitor, or to withhold a ready testimony to another's worth. Unflinching in the maintenance of what he conceived to be sound principles, he was yet diffident of his own judgment, and never conducted argument beyond the limits of the most delicate courtesy.

In professional intercourse he was ever ready to screen his brethren from any discredit which the unavoidable imperfection of our art might involve; and delighting to hear of the merited success of others, was incapable of that illiberal feeling which could apprehend in another's progress any impediment to his own. In relation to patients it was obvious that his interest and ease were regarded by him as secondary objects, and that his anxious thoughts were directed not so much to pecuniary gain as to the improvement and benevolent application of medical resources. His conscientious and sustained attention to his patients' condition seldom failed to secure their confidence, whilst his genuine sympathy, and regard to their mental and bodily welfare, won the friendship of all those who knew how to appreciate excellence. His feeble health and ultimate retirement were indeed mainly attributable to a sense of inability to realize the high estimate which he held of the duties of a physician.

But amidst conscious imperfection his hopes rested on the cardinal doctrines of Christianity; the light of which guided his course. That light no assumption of superiority tempted him to obtrude, but no timid reserve induced him to hide. The description he once wrote of another may be fairly applied to himself: "The doctrines of Christianity were the foundation of his hopes, and its holy precepts the invariable rule of his conduct; and confidence in the religion of the gospel proved his solace in affliction and support in death."

Dr. Burder has left no family; but his widow, long the soothing associate of his weakness and sufferings, awaits in calm resignation the period of reunion to one who although withheld by delicate health from attaining the elevated position which his talents and acquirements might otherwise have secured, yet well deserves an honoured place among the worthies of our profession.

